# Design and preliminary evaluation of a newly designed patient-friendly discharge letter – a randomized, controlled participant-blind trial

**DOI:** 10.1186/s12913-021-06468-3

**Published:** 2021-05-12

**Authors:** Christian Smolle, Christine Maria Schwarz, Magdalena Hoffmann, Lars-Peter Kamolz, Gerald Sendlhofer, Gernot Brunner

**Affiliations:** 1 Division of Plastic, Aesthetic and Reconstructive Surgery, Department of Surgery, Medical University of Graz, Auenbruggerplatz 29/4, 8036 Graz, Austria; 2 Research Unit Safety in Health, Division of Plastic, Aesthetic and Reconstructive Surgery, Department of Surgery, Medical University of Graz, Auenbruggerplatz 29/4, 8036 Graz, Austria; 3 Division of Endocrinology and Diabetology, Department of Internal Medicine, Medical University of Graz, Auenbruggerplatz 15, 8036 Graz, Austria

**Keywords:** Healthcare quality improvement, Comparative effectiveness research, Surveys, Patient-centred care, Patient education

## Abstract

**Background:**

Low health literacy has been associated with poor health outcome and impaired use of healthcare services. The hospital discharge letter represents a key source of medical information for patients and can be used to address the problem of low health literacy. The aim of this project was to develop and evaluate a new, patient-directed, version of the discharge letter.

**Methods:**

Based upon two conventional discharge letters (CDL; one surgical and one medical letter), two new, patient-friendly discharge letters (PFDL) were designed following 5 key principles: short sentences, few abbreviations, large font size, avoidance of technical terms and no more than 4 pages length. Medical undergraduates were randomized into two blinded groups (CDL, PFDL) and asked to assess the assigned letter for the 3 domains structure, content and patient-friendliness. Subsections were rated on a 6-point Likert scale (1 = completely agree, 6 = completely disagree), the results of the survey were compared using the Mann-Whitney-U-Test with a p < 0.05 being the level of significance.

**Results:**

In total, 74 undergraduates participated in this study. PFDL (35 participants) were rated significantly better than CDL (39 participants) regarding structure (median 1 vs. 2, p = 0.005), content (1 vs. 3, p < 0.001) and patient-friendliness (2 vs. 6, p < 0.001). Of all 17 subsections, PFDL were rated significantly better in 12 cases, and never worse than CDL.

**Conclusions:**

PFDL were rated significantly better than their CDL counterparts. Medical undergraduates were considered the ideal cohort, not being medical lays and yet unbiased regarding everyday clinical practice procedures. Further tests evaluating the impact of the PFDL on patient comprehension and health literacy are necessary.

**Supplementary Information:**

The online version contains supplementary material available at 10.1186/s12913-021-06468-3.

## Introduction

High health literacy can be seen as one of the biggest assets in patients receiving healthcare, whereas low health literacy can be pictured as a clinical risk [1]. Concomitantly, low health literacy has consistently been associated with poorer health outcome as well as poorer use of health services, reflected in increased hospitalisations, greater emergency care use, lower use of mammography, lower receipt of influenza vaccine, poorer ability to interpret health messages and increased mortality among seniors [2, 3]. It could be demonstrated that trust in health information strongly depends on the individual level of health literacy: People with lower health literacy tend to use and trust sources with limited quality of health information, such as social media, celebrity blogs, television or information provided by pharmaceutical companies and put low trust in information from physicians. In contrast, people with high health literacy use and put higher trust in higher-quality information provided by physicians or healthcare websites [4]. To deliver optimal service, health care providers have to pursue a bilateral approach: on the one hand improving patients’ health literacy through patient education and on the other hand providing information suitable to the patients’ health literacy level. The former task needs a community-based approach [5], while the latter can be accomplished by individual healthcare providers through face-to-face conversation or adequate presentation of medical information [6].

During hospitalisation a patient receives a variety of information from different sources – physicians, nurses, therapists and social workers might inform the patient about diagnostic findings, remind of medications, give instructions for exercises or discuss the patient’s post-discharge rehabilitation program. Ideally, a discharge letter should contain all of this information as a take-home message for the patient. In addition, adequate presentation and quality of the information, as well as timely delivery of the discharge letter to ensure unbroken information between hospitals, patients and general practitioners is of utmost importance [7]. Former discharge letter interventions intended to improve information flow between hospitals and general practitioners [8–10]. Recently, with the evolving concept of patient-centred communication, discharge information increasingly aims to address the patient directly, also with the help of modern technology such as reminders sent to the patient via short message service [11–13], or letters sent electronically thus entirely replacing the conventional paper-based transmission [14, 15]. It could be demonstrated that patient-directed discharge information significantly improved patient understanding of the reason for hospitalisation and post-discharge recommendations [6].

Started by the Executive Department for Quality and Risk Management at the University Hospital Graz, Austria, the aim of the so-called “GO-SAFE” project was to improve the discharge information. In this case, the discharge letter should satisfy both the needs of physicians and patients for effective discharge communication. Based upon the following key principles, the discharge letter was redesigned: The discharge letters should (i) be as short as possible, (ii) be written in a simple and comprehensible way (abbreviations, structure, headings), (iii) provide the next treating physician (general practitioner/specialist) with all necessary information – i.e. no information is lost due to simplicity of formulation. In addition, the discharge letter should comply with the standards set by ELGA (“Elektronische Gesundheitsakte” – Austrian Electronic Health Records; established by the Austrian ministry of health in 2015 with the aim to make patients’ health data digitally available for healthcare providers) to become an integrative part of patients’ electronic health records. Previous steps in the process of GO-SAFE included risk analysis of information loss during hospital discharge [7], a survey among 1060 physicians towards content and target groups of discharge summaries [16], as well as analysis of current discharge letters to identify possible deficiencies [17]. As the target groups of a new discharge letter are patients as well as physicians and therapists, a multi-level analysis was deemed necessary. In the current process step of GO-SAFE, the prototype of the new discharge letter was evaluated by medical students.

## Materials and methods

### Concept of the new discharge letter

As a first step, an analysis of structure, content, unsafe abbreviations, and completeness of 100 of discharge letters was done [17]. Based on this knowledge, two conventional discharge letters (CDL), one surgical, one medical, that were randomly selected from the sample of 100 discharge letters from surgical and medical specialties, two new, patient-friendly discharge letters (PFDL) were created according to the guidelines of the final version of the HL7 Implementation Guide for discharge letters for ELGA [18]. According to these guidelines, a conforming discharge letter should contain a minimum of mandatory information based upon following sub-headings:

- Reason for admission.
- Discharge diagnosis, secondary diagnoses.
- Diagnostic and therapeutic measures.
- Recommended medication – including tradename, active substance, route of administration, prescription and dosage.
- Therapy recommendations.
- Discharge summary.
- Information on possible allergies, intolerances and individual risk factors (e.g. miscellaneous implants).
- Summary of diagnostic findings.
- Anamnesis (optional)..

In addition, the results of a survey conducted previously as part of GO-SAFE among Austrian physicians were taken into consideration. Accordingly, > 95 % of the survey participants considered information on diagnosis, treatment, prescription of medication, recommendation on further treatment as well as information on control visits and follow-up appointments as compulsory elements of an adequate discharge letter [16].

Since previous studies indicated poor comprehension of medical terms among patients and its negative impact on compliance [19], special attention was also paid to patient-friendly formulation of the document, by complying with few general principles, adapted from the “Guidelines for easy-to-read materials” [20]:

- Use of short sentences (12–15 words).
- Restrictive use of abbreviations, and if used explanation upon first mentioning.
- Whenever possible avoidance of technical terms.
- Font size at least 12.
- No more than 4 pages.

In line with the aforementioned considerations the PFDL was designed by the first author and thereafter checked for accuracy of information and medical content and approved by four independent auditors (two clinicians, one quality manager, one quality manager assistant).

### Participants and assessed parameters

Medical students were chosen because of their special role in the context of health literacy. Depending on their year of training they do not yet have profound medical knowledge, but they already have basic knowledge of individual terms. Consequently, like patients, they are already experts in certain specialist areas and are therefore ideal partners in a process-based approach for the further development and expansion of the evidence. Participants were third-year medical students who were recruited from a mandatory lecture on quality and safety in healthcare. The participants were informed about background and aim of the study, but did not know, which version of the discharge letter (CDL or PFDL) they would ultimately be given to asses. Informed consent was obtained from all participants prior to handing out the questionnaires. Assessed demographic parameters included gender, age and study semester.

### Questionnaire

The questionnaire was in German language and consisted of 4 subsections: (i) participant information, (ii) structure (3 questions), (iii) content (11 questions), and (iv) patient-friendliness (3 questions) of the discharge letter. Additionally, there was an open section for personal feedback from each candidate. All questions concerning the letter could be answered on a 6-point Likert-scale (1 = completely agree, 6 = completely disagree). A translated version of the questionnaire is provided in Table 1.

**Table 1:** Questionnaire (translated from German). CDL = conventional discharge letter, PFDL = patient-friendly discharge letter. Section i) included only questions on demographic data and is not shown. ES = effect size (Cohen’s d), * = small effect (d > 0.2), ** = moderate effect (d > 0.5), *** = large effect (d > 0.8). Values are given in medians (interquartile range, IQR), significant p-values in bold

Questions (translated from German)	CDL	PFDL	*ES (d)*	p-value
*Section i, (demographic data, not shown)*				
*Section ii, structure – overall*	2 (1–2)	1 (1–2)	0.50**	**0.005**
2.1 Is the layout clear?	2 (1–2)	1 (1–2)	0.51**	**0.042**
2.2 Is the structure comprehensible?	2 (1–2)	1 (1–2)	0.11	0.545
2.3 Does the content justify the length of the document?	2 (1–3)	1 (1–2)	0.74**	**0.001**
*Section iii, content – overall*	3 (2–3)	1 (1–2)	1.26***	**< 0.001**
3.1 Are the used abbreviations clear?	3 (2–4)	2 (1–4)	0.24*	0.216
3.2 Are abbreviations explained?	6 (5–6)	2 (1–4)	2.13***	**< 0.001**
3.3 Is the referral letter phrased in a comprehensible way?	2 (1–3)	1 (1–2)	0.43*	**0.006**
3.4 Is the chronological sequence of events during hospital stay presented in a conclusive manner?	2 (1–3)	2 (1–2)	0.42*	0.130
3.5 Are main and secondary diagnoses clearly evident?	1 (1–2)	1 (1–1)	0.35*	0.278
3.6 Are the reasons for the therapeutic approach during hospital stay comprehensible?	2 (1–3)	1.5 (1–3)	0.43*	0.090
3.7 Are therapy recommendations and goals of rehabilitation clear?	2 (1–5)	1 (1–2)	0.69**	**0.004**
3.8 Are the therapeutic steps taken described in detail and comprehensible?	2 (1–4)	2 (1–2)	0.72**	**0.008**
3.9 Is the recommended medication described in detail, including trade name, active ingredient, dosage and route of administration?	2 (1–4)	1 (1–1)	1.01***	**< 0.001**
3.10 Is there information concerning possible allergies?	6 (6–6)	1 (1–1)	4.40***	**< 0.001**
3.11 Is contact information of a doctor provided for possible queries?	3 (1–5)	1 (1–2)	1.00***	**< 0.001**
*Section iv, patient-friendliness – overall*	6 (5–6)	2 (1–2)	2.72***	**< 0.001**
4.1 Would a medical layperson be able to understand the content of the referral letter?	6 (5–6)	3 (2–4)	1.94***	**< 0.001**
4.2 Would the indication of the prescribed medication be comprehensible for a medical layperson?	6 (5–6)	1 (1–2)	3.23***	**< 0.001**
4.3 Would a medical layperson be able to deduce necessary further diagnostic or therapeutic measures from the referral letter?	4 (2–5)	1 (1–2)	1.31***	**< 0.001**
*Overall score*	3 (2–3)	1 (1–2)	1.38***	**< 0.001**

### Study design

**Fig. 1:**
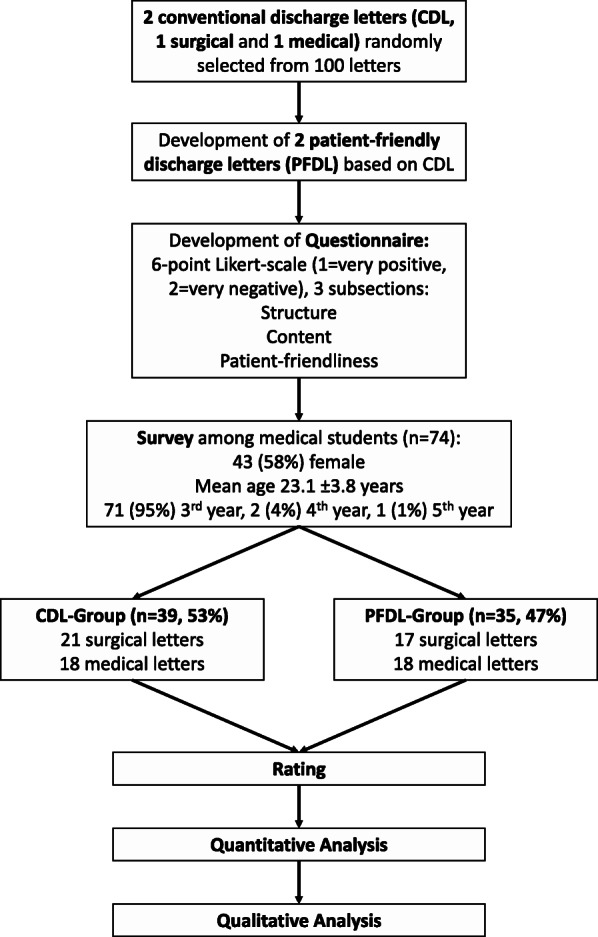
Flow chart showing the study design and general results. PFDL were designed based upon CDL (one surgical, one medical) and a questionnaire was developed. Medical undergraduates were given the letters to rate them for structure, content and patient-friendliness. Of note, each undergraduate received only one letter, either surgical or medical in its PFDL or CDL form

The study was concepted as a randomized controlled participant-blind trial. After informed consent, the participants were randomly assigned to one of the two study groups (50 % SCL/50 % PFDL by random), one assessing the CDL and one the PFDL. Consequently, each participant received one discharge letter, either surgical or medical in its CDL or PFDL form to asses. Throughout the survey, participants were allowed to ask questions concerning the questionnaire, however not concerning the discharge letters to preclude any observer bias. Figure 1 is a flow chart demonstrating the study design.

### Statistical analysis

Statistical analysis was done with SPSS 24.0 for Windows (IBM Inc., Armonk, NY, USA). Total overall median scores and median scores with interquartile ranges (IQR) of the subsections were calculated. Univariate inductive analysis comprised the Chi²-test for dichotomous variables and Student’s T-test for continuous parameters. Mann-Whitney-U-test for independent samples was used to compare ordinal variables between the two groups. Cohen’s d was used to calculate effect size (ES). A d ≥ 0.2 was considered as small, a d ≥ 0.5 as moderate, and a d ≥ 0.8 was considered as large effect. A p-value below 0.05 was considered as statistically significant. The personal feedback of the participants was analysed qualitatively. The feedback was stratified into positive, negative and neutral by a single reviewer. The classification was then cross-checked and approved by two additional reviewers. Feedback was deemed positive when aspects of and/or the entire letter were deemed suitable for patients, neutral, if some aspects of the content were deemed suitable and some not suitable for patients and negative if the entire letter and/or certain aspects were deemed unsuitable for patients.

## Results

### General demographics

Of a total of 125 candidates, 74 students completed the survey (response rate 59.2 %). There were 43 (58 %) female participants, and mean age was 23.1 ± 3.8 years. Seventy-one (95 %) students were in their 3rd year of medical studies, while 2 (3 %) were in their 4th, and 1 (2 %) in his 5th year. Thirty-nine (53 %) were in the CDL, 35 (47 %) in the PFDL group. There were 38 surgical discharge letters (21 CDL, 17 PFDL) and 36 medical discharge letters (18 CDL, 18 PFDL, see Fig. 1). There was no significant difference regarding gender, age, year of medical studies or the distribution of surgical/medical letters between the two study groups (all p > 0.05). Raw data of the survey results is provided in additional file 1.

### Results of the survey

Overall, the PFDL group rated the discharge letters significantly better than the CDL group (median [IQR]: 1 [1-2] vs. 3 [2-3], RI = + 2, p < 0.001). Separate comparison of subsections yielded similar results, which were median 1 (IQR 1–2) vs. 2 (1–2) for section ii (structure, Cohen’s d = 0.5, p = 0.005), 1 (1–2) vs. 3 (2–3) for section iii (content, d > 0.8, p < 0.001) and 2 (1–2) vs. 6 (5–6) for section iv (patient-friendliness, d > 0.8, p < 0.001) in the PFDL and CDL group, respectively. When questions were analysed one by one, the PFDL group rated their letters significantly better in 12 cases and never worse than the CDL group. In questions with significant difference, Cohen’s d indicated small ES in one case, moderate ES in four, and large ES in seven cases (for detailed results see Table 1).

Additionally, questions with the largest ES were identified in each section. In section ii (structure), ES was greatest for question 2.3 (“Does the content justify the length of the document?”, d = 0.74, moderate ES). In section iii (content), ES was greatest for question 3.10 (“Is there information on possible allergies?”, d = 4.40, large ES). This is unsurprising, as information on possible allergies was entirely missing in both original discharge letters, but was given in the new letters. In section iv (patient-friendliness), question 4.2 (“Would the indication of the prescribed medication be comprehensible for a medical layperson?”) had the largest ES (d = 3.23, large effect).

### Subgroup analysis

The subgroup analysis revealed that students given the surgical PFDL rated their versions significantly better than the surgical CDL group in 11 cases, whereas students given the medical PFDL rated their versions significantly better than the medical CDL group in nine cases. Section by section, participants with the surgical PFDL rated their letters significantly better than the surgical CDL group in all sections (ii: p = 0.005, d = 0.89; iii: p = 0.006, d = 1.01; iv: p < 0.001, d = 2.53) and overall (p < 0.001, d = 1.24), whereas the medical PFDL group rated their letters significantly better than the medical CDL group overall (p < 0.001, d = 1.50) and in sections iii (content, p < 0.001, d = 1.50) and iv (patient-friendliness, p < 0.001, d = 3.14), however not in section ii (structure, p = 0.988, d = 0.09) for detailed results see Table 2).

**Table 2:** Results of the subgroup analysis for surgical and medical discharge letters, comparison between CDL and PFDL. Section 1 was demographic data and is not shown, ES = effect size (Cohen’s d), * = small effect (d ≥ 0.2), ** = moderate effect (d ≥ 0.5), *** = large effect (d ≥ 0.8). Values are given in medians (IQR), significant p-values in bold

	*Surgical*				*Medical*			
Questions	**CDL**	**PFDL**	***ES (d)***	**p-value**	**CDL**	**PFDL**	***ES (d)***	**p-value**
*Section i, (demographic data, not shown)*								
*Section ii, structure – overall*	2 (2-2.5)	1 (1–2)	0.89***	**0.005**	1 (1–2)	1 (1–2)	0.09	0.988
2.1 Is the layout clear?	2 (1.5-3)	1 (1-1.75)	0.99***	**0.004**	1 (1–2)	1.5 (1–2)	0.15	0.719
2.2 Is the structure comprehensible?	2 (2–2)	1 (1–2)	0.71**	**0.039**	1 (1–2)	1 (1–3)	0.63**	0.214
2.3 Does the content justify the length of the document?	2 (1–3)	1 (1–2)	0.49*	0.152	2 (1–3)	1 (1–1)	1.04***	**0.004**
*Section iii, content – overall*	2 (2–3)	1 (1–2)	1.01***	**0.006**	3 (1.75-4)	1 (1–2)	1.50***	**< 0.001**
3.1 Are the used abbreviations clear?	2 (1-3.5)	3 (2–5)	0.30*	0.352	3 (2.75-5)	2 (1–2)	0.92***	**0.003**
3.2 Are abbreviations explained?	6 (5–6)	2 (1–4)	1.52***	**< 0.001**	6 (6–6)	1.5 (1–3)	3.06***	**< 0.001**
3.3 Is the referral letter phrased in a comprehensible way?	2 (2–3)	1 (1–3)	0.17	0.170	2 (1–3)	1 (1–2)	0.76**	0.051
3.4 Is the chronological sequence of events during hospital stay presented in a conclusive manner?	2 (1.5–3.5)	2 (1–3)	0.43*	0.308	2 (1–3)	1.5 (1–2)	0.38*	0.424
3.5 Are main and secondary diagnoses clearly evident?	1 (1–1)	1 (1–1)	0.02	0.885	1 (1–3)	1 (1–1)	0.70**	0.171
3.6 Are the reasons for the therapeutic approach during hospital stay comprehensible?	2 (1–3)	2 (1–3)	0.49*	0.280	2 (1-3.25)	1 (1-2.25)	0.40*	0.279
3.7 Are therapy recommendations and goals of rehabilitation clear?	2 (1-3.5)	1 (1–1)	0.98***	**0.006**	3 (1–5)	1.5 (1–3)	0.55**	0.161
3.8 Are the therapeutic steps taken described in detail and comprehensible?	2 (1.5–3.5)	1 (1–2)	0.71**	**0.036**	2.5 (1-4.25)	2 (1–3)	0.73**	0.118
3.9 Is the recommended medication described in detail, including trade name, active ingredient, dosage and route of administration?	1 (1-4.5)	1 (1–1)	0.85***	**0.026**	2.5 (1.75-4)	1 (1–1)	1.22***	**0.001**
3.10 Is there information concerning possible allergies?	6 (5–6)	1 (1–1)	4.60***	**< 0.001**	6 (6–6)	1 (1–1)	4.07***	**< 0.001**
3.11 Is contact information of a doctor provided for possible queries?	2 (1–4)	1 (1–2)	0.72**	**0.030**	4 (1.5-6)	1 (1–1)	1.30***	**0.001**
*Section iv, patient-friendliness – overall*	5 (4–6)	2 (1–2)	2.53***	**< 0.001**	6 (5–6)	2 (1–3)	3.14***	**< 0.001**
4.1 Would a medical layperson be able to understand the content of the referral letter?	6 (5–6)	2 (2–4)	1.69***	**< 0.001**	6 (5–6)	3 (2–4)	2.19***	**< 0.001**
4.2 Would the indication of the prescribed medication be comprehensible for a medical layperson?	6 (4.5-6)	1 (1–2)	2.82***	**< 0.001**	6 (5.75-6)	2 (1-2.25)	4.18***	**< 0.001**
4.3 Would a medical layperson be able to deduce necessary further diagnostic or therapeutic measures from the referral letter?	3 (2.5-5)	2 (1–2)	1.52***	**< 0.001**	4 (2-5.25)	1 (1-2.25)	1.18***	**0.001**
*Overall*	2 (2–3)	1 (1–2)	1.24***	**< 0.001**	3 (2–4)	1 (1–2)	1.50***	**< 0.001**

There was no significant difference regarding mean age, gender or year of study between the PFDL and CDL groups within the surgical and medical subgroup. Furthermore, there were also no significant differences between the surgical and medical subgroups regarding demographic parameters (see Table 3).

**Table 3:** Subgroup analysis of demographic data

Parameter	Surgical CDL	Surgical PFDL	p-value	Medical CDL	Medical PFDL	p-value	Surgical CDL + PFDL	Medical CDL + PFDL	p-value
Age, mean (± SD)	22.7 (± 1.7)	22.3 (± 0.9)	0.382	23.7 (± 7.0)	23.6 (± 2.2)	0.940	22.5 (± 1.4)	23.7 (± 5.2)	0.213
Female gender, n (%)	10 (48 %)	12 (71 %)	0.154	11 (61 %)	10 (56 %)	0.735	22 (58 %)	21 (58 %)	0.970
Year of study, n (%)			0.112			0.324			0.583
3rd year	21 (100 %)	15 (88 %)		18 (100 %)	17 (94 %)		36 (94 %)	35 (97 %)	
4th year	-	1 (6 %)		-	1 (6 %)		1 (3 %)	1 (3 %)	
5th year	-	1 (6 %)		-	-		1 (3 %)	-	

### Results of open-answer question

For the written feedback given on open answer questions are listed in additional file 2 .

In the CDL group, 9 of 39 participants (23 %) gave written feedback concerning the properties of the discharge letters. Thereof feedback was positive in 1 case, neutral in 1 and negative in 7 cases. Thorough analysis of the given answers revealed that the liberal use of abbreviations was a major concern and cause for negative feedback in 5 cases.

In the PFDL group, 11 of 35 participants (31 %) gave written feedback. Feedback was considered positive in 6 cases, neutral in 2 and negative in 3 cases. Among the positive feedback, three main categories could be identified: structure of the discharge letter, explanation of technical terms and the new list for drug prescriptions.

## Discussion

In the present study we prospectively compared 2 newly designed PFDL (one surgical, one medical) to 2 CDL (one surgical, one medical) taken from current clinical practice by means of a questionnaire study done with 3rd -year medical undergraduates. Overall, the PFDL were rated significantly more positive and received better feedback than the CDL, regardless of specialty (surgical/medical).

In line with the awareness that health literacy is one of the most important assets in patient-centred health care [21], and considering the fact that discharge letters in their traditional form can pose a hindrance in effective and efficient communication between patients and healthcare providers [6, 22, 23], the aim of this so-called “Go-SAFE” project was – inter alia – to develop a PFDL to be ultimately used in clinical practice. Thereby, the new letter should satisfy some general principles of patient-centred communication: short sentences, few abbreviations (explained upon use), few technical terms, adequate font size (12 pts.) and at most 4 pages. At the same time the letter should also comply with ELGA-standards – this in order to become an integrative part of the electronic health records in Austria. In this respect the present study was more or less a proof of principal, i.e. an objective assessment of whether a newly designed letter would improve information flow in the eyes of unbiased (since not involved into daily medical practice), and yet reasonable candidates from a medical perspective.

Overall, the PFDL were rated significantly better than their original counterparts. In the “structure” section, participants presented the new letters were significantly more pleased with the layout as well as the content relative to the document size. However, the rating of the actual structure of the document, i.e. the sequence of headings and sub-headings did not differ significantly from that of original letters, despite extensive changes had been made there – this might indicate that the sequence of information is irrelevant as long as it is provided in comprehensible manner. The biggest effects were observed in the “content” section: Firstly, abbreviations were explained and clear to the participants significantly more often. The potential danger of miscommunication evolving from medical abbreviations has been investigated by Shilo and Shilo, who found that medical and orthopaedic senior physicians were unfamiliar with the meaning of 14 and 25 %, of medical abbreviations, respectively, used in discharge letters of junior colleagues [24, 25]. Furthermore, the new letters were considered to be written in a significantly more comprehensible way, provide significantly more precise information on therapeutic steps taken as well as goals of rehabilitation and recommended medication and were more often seen to provide contact details of treating physicians and/or therapists. Although this information was at least partially provided in CDL, it seems as if already the new arrangement of information helped to attract the reader’s attention and make it instantly visible. All 3 subsections of the section “patient-friendliness” – the main objective of the study – were rated significantly better by the PFDL group. More specifically, PFDL participants considered it more likely that laypersons would be able to understand the content of the letter, understand the indications for their medication and would easily be able to deduce all necessary information on post-discharge therapies from the document. According to a study by Albrecht et al., prevalence of non-comprehension of discharge instructions was as high as 27 % for medication, 48 % for exercise plans and 50 % for dietary instructions [26]. Non-comprehension has furthermore been associated with high complexity of information besides low health literacy [27]. Since 3rd -year undergraduates judged the PFDL easier to understand it can be assumed that the applied measures helped to effectively decrease complexity of discharge instructions.

Qualitative analysis of the written feedback further confirmed the results obtained from the questionnaire. For example, while the CDL group criticised the liberate use of abbreviations, the PFDL group appraised the restrictive use of abbreviations and their immediate explanation extremely positive. Furthermore, the written feedback provided important insights into key aspects a medical layperson might expect from a discharge letter. It was immediately noticed when information on medication or post-discharge measures was not provided, whereas the presence of this information was the cause for positive feedback several times. It has been shown that more than 80 % of patients feel they receive much irrelevant and too little immediately relevant information upon discharge [28]. Therefore, it is important to keep in mind that a discharge letter should contain very practical information on what to DO and, more importantly, what NOT TO DO – and even if no further diagnostic or therapeutic steps are necessary this should be clarified.

### Limitations

Since the study cohort consisted of medical undergraduates, it would be premature trying to translate the results directly to clinical practice. However, the study served as a proof-of-principle and pointed out some important issues to keep an eye on when re-thinking the concept of the conventional discharge letter. At this step of the process no patients were included into the evaluation. A survey including patients as well as stakeholders is planned as the logical next step succeeding the present study.

## Conclusions

Compared to original counterparts used in clinical practice, the patient-friendly discharge letter achieved better acceptance and more positive feedback among medical undergraduates. These results can primarily be attributed to changes in document structure (less abbreviations, explanation of medical technical terms, bigger font size), content (clear post-discharge instructions) and improved patient-friendliness. Further studies concerning the impact of these measures on comprehension of discharge information among medical lays and patients are needed.

### Trials registration

This study has been registered as clinical trial (ClinicalTrials.gov Identifier: NCT04628728).

## Supplementary Information

**Additional file 1.**

**Additional file 2.**

## Data Availability

The dataset supporting the conclusions of this article is included within the article (and its additional files). The dataset can be obtained from the corresponding author upon reasonable request. Contact details: Gerald Sendlhofer, PD Mag. Dr., Division of Plastic, Aesthetic and Reconstructive Surgery, Department of Surgery, Medical University of Graz, Auenbruggerplatz 29/4, 8036 Graz, Austria, Email: gerald.sendlhofer@medunigraz.at, Telephone: +43 316 385 14,685.

## Abbreviations

CDL: Conventional discharge letter
ELGA: “Elektronische Gesundheitsakete” – Austrian Electronic Health Records
ES: Effect size
IQR: Interquartile range
IRB: Institutional review board
PFDL: Patient-friendly discharge letter
