# Supplementary material for: Design and preliminary evaluation of a newly designed patient-friendly discharge letter – a randomized, controlled participant-blind trial
Source: BMC Health Serv Res. 2021 May 12;21:450. doi: 10.1186/s12913-021-06468-3 (PMC8114527; doi:10.1186/s12913-021-06468-3)
Supplement: Supplementary file 2 — Additional file 2. [file 12913_2021_6468_MOESM2_ESM.docx]

Additional file 2: Table with results of open-answer questions and their qualitative analysis.

| Conventional discharge letter | Patient-friendly discharge letter |
| --- | --- |
| **Positive** |  |
| *Very elaborate, provides good overview* | *Through information in brackets given in “non-medical language very easily understandable* |
|  | *I believe the letter is good. It is especially helpful that the medications have been listed as tables* |
|  | *The letter is written in a comprehensible way. Very detailed, good structure.* |
|  | *Good structure. Explanation in plain language at the end of each letter and additional information on medication are good.* |
|  | *Explanation of medical terms is very helpful, good solution!* |
|  | *Translation of medical terms into German language is certainly useful.* |
| **Neutral** |  |
| *Feedback was given based on the presumption that the letter should be comprehensible for the patient in the first place* | *Useful: Highlighting of appointments and important medication. Not useful: some terms probably not understandable.* |
|  | *Probably not all medical terms are described in a comprehensible way. Otherwise, good structure, especially the “to-do-list” for the patient is useful.* |
| **Negative** |  |
| *Personally, I believe that the term NSAID should be avoided in referral letters. Lays do not know what that is, while over-the-counter medications [e.g. NSAIDs] can be found in almost any household. Furthermore, many abbreviations may contribute to the patient’s disconcertion* | *There is little time for writing such an extensive letter in daily clinical practice!* |
| *It is likely that the patient takes most medications on a regular basis, therefore it might be superfluous to list them all up in the letter.* | *More information on the patient’s medication would be desirable.* |
| *Therapy recommendations are missing – even if e.g. no drugs were prescribed, this should be mentioned in the discharge letter. Abbreviations in the list of diagnoses are o.k., however, when abbreviations are used, they should at least be explained in the text.* | *Better explanation of medical terms, information on consequences of results of diagnostic test is desirable.* |
| *The letter is written as to meet the general practitioner’s needs, but is not sufficiently extrapolated to be understood by lays.* |  |
| *I believe it would be best not to use any abbreviations at all, as they are not only hard to understand, but are sometimes even identical although they refer to different terms.* |  |
| *Jumble of numbers and words* |  |
| *Too many technical terms and abbreviations and therefore not comprehensible for lays* |  |
